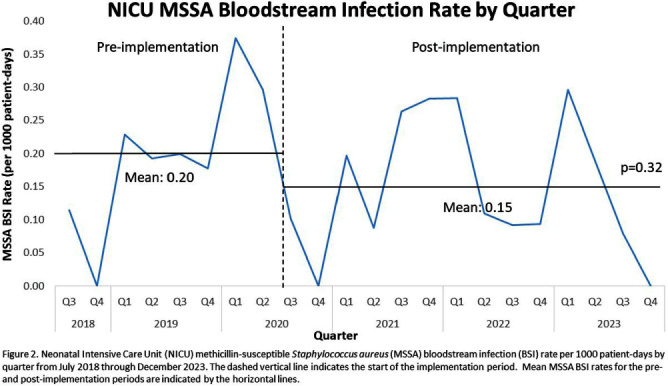# S. aureus Surveillance and Decolonization Associated with Decreased MRSA, but not MSSA, Infections in the Neonatal ICU

**DOI:** 10.1017/ash.2024.273

**Published:** 2024-09-16

**Authors:** Patrick Reich, Geoffrey Ikpeama, Angela Niesen, Ashley Lloyd, Stephanie Fritz

**Affiliations:** Washington University School of Medicine; BJC Healthcare - St. Louis Children’s Hospital; Washington University in St. Louis School of Medicine

## Abstract

**Background:** Invasive Staphylococcus aureus infections cause significant morbidity and mortality in neonatal intensive care unit (NICU) infants.1 Colonization (asymptomatic carriage in the nose, skin, or gut) is a risk factor for subsequent invasive infection (e.g., pneumonia, bone infections, bloodstream infections, etc.). Active surveillance and decolonization measures for S. aureus-colonized infants have been associated with decreased invasive infection rates. 2-4 **Methods:** A methicillin-resistant S. aureus (MRSA) surveillance and decolonization program, consisting of admission and weekly MRSA nasal cultures followed by intranasal mupirocin plus chlorhexidine baths for colonized infants, was implemented in our level IV NICU with 150 beds in 2006.5 Due to poor compliance with decolonization protocols5, existing practices were reviewed and multiple interventions to increase compliance were implemented in 2018. These renewed efforts included revision of the existing MRSA decolonization protocol, updating the associated electronic medical record order set, re-education of unit staff, and weekly review by the Infection Prevention (IP) and NICU leadership teams to ensure the decolonization protocol was followed for newly colonized infants. Mean MRSA bloodstream infection (BSI) rates were calculated quarterly pre- (January 2014-December 2017) and post- (January 2018-December 2023) implementation of renewed efforts and compared via unpaired t-test. In July 2020 a similar methicillin-susceptible S. aureus (MSSA) surveillance and decolonization program was implemented with an associated revision of existing documents, education campaign, and weekly review of infants with new MSSA colonization. Mean MSSA BSI rates pre- (July 2018-June 2020) and post- (July 2020-December 2023) implementation were compared via unpaired t-test. **Results:** Renewed implementation of MRSA surveillance and decolonization was associated with a sustained decrease in the mean MRSA BSI rate (Figure 1): 0.10 per 1000 patient-days pre-implementation, 0.03 post-implementation (p=0.02). Following implementation of MSSA surveillance and decolonization, there was no statistically significant change in the mean MSSA BSI rate (Figure 2): 0.20 per 1000 patient-days pre-implementation, 0.15 post-implementation (p=0.32). **Conclusions:** Implementation of a robust MRSA surveillance and decolonization program in the NICU was associated with a sustained decrease in invasive MRSA infections. No change in invasive MSSA infection rates was observed following implementation of a similar protocol for MSSA. Additional research is needed to better understand the role of MSSA surveillance and decolonization in the NICU.

**References:** 1. Ericson, J.E., et al., JAMA Pediatr, 2015. 2. Popoola, V.O., et al., ICHE, 2016. 3. Kotloff, K.L., et al., Pediatrics, 2019. 4. Voskertchian, A., et al., ICHE, 2018. 5. Reich, P.J., et al. Clin Microbiol Infect, 2016.

**Figure:**
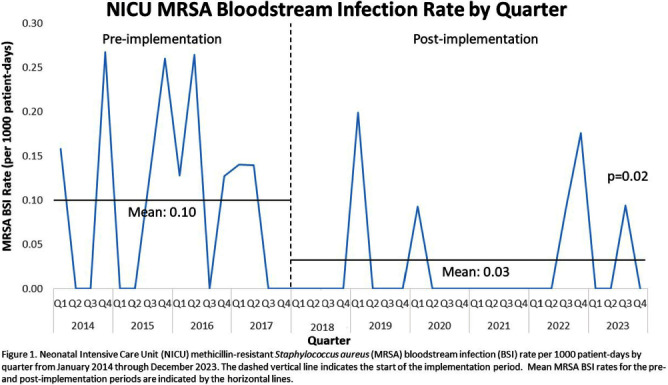

**Figure:**